# Tailoring survivorship care for older long-term survivors of breast and colorectal cancer

**DOI:** 10.1093/jncics/pkag026

**Published:** 2026-05-11

**Authors:** Samuel X Stevens, Ashanya Malalasekera, Sim Yee Tan, Janette L Vardy

**Affiliations:** Faculty of Medicine and Health, The University of Sydney, Sydney, NSW, Australia; Sydney Cancer Survivorship Centre, Concord Repatriation General Hospital, Concord West, NSW, Australia; Department of Oncology, Queen’s University, Kingston, ON, Canada; Faculty of Medicine and Health, The University of Sydney, Sydney, NSW, Australia; Sydney Cancer Survivorship Centre, Concord Repatriation General Hospital, Concord West, NSW, Australia; Department of Medical Oncology, Bankstown-Lidcombe Hospital, Revesby, NSW, Australia; Supportive Care, Chris O’Brien Lifehouse Australia, Camperdown, NSW, Australia; Faculty of Medicine and Health, The University of Sydney, Sydney, NSW, Australia; Sydney Cancer Survivorship Centre, Concord Repatriation General Hospital, Concord West, NSW, Australia; Faculty of Medicine and Health, The University of Sydney, Sydney, NSW, Australia; Sydney Cancer Survivorship Centre, Concord Repatriation General Hospital, Concord West, NSW, Australia

Care for older adult cancer survivors must strike a delicate balance. In the first 5 years following treatment, clinicians must be attentive to the late effects of cancer and its treatment, including the risk of early recurrence, while recognizing that many older survivors face substantial competing risks to life and health unrelated to their cancer. This reality becomes ever more salient as time passes since the index cancer diagnosis. In the ensuing years, survivorship care for many older people unfolds against a backdrop of multimorbidity, functional decline, and engagement with the healthcare system for nononcologic conditions.^1^^,^^2^ Understanding the absolute risk of late cancer recurrence in the context of these competing demands is essential to designing survivorship care that is proportionate, effective, and minimally disruptive to the lives of older adults.

In this issue of the Journal, Yasin et al.^3^ provide important data on the risk of long-term recurrence of colorectal cancer (CRC) and breast cancer in a cohort of more than 30 000 adults aged 66 years or older who were considered well enough to undergo definitive treatment for early CRC and breast cancer. The cohort was identified from the Surveillance, Epidemiology, and End Results Program–Medicare database using a validated algorithm. Patients were diagnosed between 2003 and 2011 and remained recurrence free for at least 5 years after diagnosis.^3^ Late recurrence was uncommon, occurring in 5.0% of breast cancer survivors, 4.4% of colon cancer survivors, and 8.0% of rectal cancer survivors, and was associated with higher stage at diagnosis and traditional clinicopathological factors. As expected in an older adult population, death from cancer-related causes was far less common than death from competing causes, occurring approximately 10-fold less frequently overall and 5-fold less frequently among survivors of rectal cancer.

These patterns of recurrence were observed despite the cohort having a relatively low documented comorbidity burden at diagnosis, with 80%-85% of participants having 2 or fewer Elixhauser comorbidities. However, 15% of participants were classified as medically frail, affirming that clinically relevant vulnerability in older cancer survivors may not be captured by comorbidity counts alone.^4^ Patients were infrequently prescribed adjuvant chemotherapy, and any treatment-related toxicities did not appear to affect the quantity of remaining life. Broadly, the study population had a high proportion of patients of non-Hispanic White ethnicity, had low area-level poverty, and maintained continuous medical insurance. Taken together, this finding suggests more favorable health status and lower cancer-related risk over the trajectory of these patients’ lives compared with the general population. The findings should invite careful consideration of how successive stages of survivorship care and cancer surveillance should be prioritized.

Cancer treatment, like all medical care, is ultimately justified by its ability to improve the length or quality of patients’ lives. Yasin and colleagues^3^ assert the need to “balance cancer surveillance with overall health management in older cancer survivors.” We agree but believe that these data go farther, raising fundamental questions about where the balance ought to be found when considering the long-term survivorship care of older adults. Person-detected and caregiver-detected survivorship care involves risk-stratified surveillance, promotion of a healthy lifestyle, and management of unmet needs. Needs include health, information, functional, psychological, and socioeconomic domains. Many of these domains and models of care delivery overlap with comprehensive geriatric care.

At present, there is limited evidence to guide survivorship care for older adult cancer survivors beyond conventional surveillance landmarks. An important exception is breast cancer, where treatment for estrogen receptor–positive disease often continues beyond 5 years, with the risk of late recurrence persisting well after 10 years^5^; extending adjuvant endocrine therapy, however, does not substantially reduce either breast cancer mortality or all-cause mortality.^6^ For many older survivors, the absolute risk of late cancer recurrence is low, and mortality is overwhelmingly driven by non–cancer-related causes. In this context, survivorship care is likely to add the greatest value when it addresses broader drivers of health, including frailty, functional decline, and comorbid medical conditions, in collaboration with primary care and appropriate specialist teams. Indeed, there is substantial overlap between guideline-based extended survivorship care and comprehensive geriatric oncological care.^7^^,^^8^ Although a variety of models exist, a feasible, equitable, and community-based approach is especially important for older cancer survivors.^9^ For many older survivors, it may be appropriate to redirect attention from cancer-directed surveillance toward treatment of competing, active health problems. Overemphasis on cancer surveillance in this population is less likely to improve outcomes and may impose unnecessary burden and harm.

Warranting special consideration is the role of surveillance imaging and procedures in detecting late recurrence of breast cancer or CRC. Currently, no cancer society guidelines recommend routine surveillance imaging beyond 5 years. Indeed, the benefits of detecting and intervening for asymptomatic recurrence is disputed even in a younger, healthier population.^10^^,^^11^ Physician adherence to guidelines, however, is highly variable. Using the numbers at risk presented in Figure 3 of Yasin et al.^3^ and the 10-year cumulative incidence estimates as well as assuming annual surveillance imaging between years 5 and 9, we calculated the yield of routine imaging. On average, approximately 79 scans would be required to detect a single recurrence in breast cancer, 86 in colon cancer, and 47 in rectal cancer. These estimates should reinforce the limited role for routine surveillance imaging in older long-term survivors, as outlined in major professional society guidelines.

Resources and efforts should instead address both mortality and morbidity by incorporating and promoting healthy lifestyles into models of survivorship care tailored to the needs of older cancer survivors. There is robust evidence that exercise is beneficial during and after treatment, particularly for older adults, with substantial improvements in physical functioning, independence in activities of daily living, mood, and a reduction in falls and fall-related injuries as well as in cognitive impairment.^12^^,^^13^ Exercise has been shown to decrease the risk of several cancers, including CRC and breast cancer.^14^ The Canadian Cancer Trials Group CO21.CHALLENGE randomized controlled trial in colon cancer provided the first level 1 evidence that a structured exercise program can substantially decrease the risk of recurrence (at a median follow-up of 7.9 years) and improve overall survival.^15^ In addition, there was an improvement in quality of life and function, with only a modest increase in musculoskeletal adverse events. There was no age limit in CO21.CHALLENGE; the median age of participants was 61 years, with an upper range of 84 years. The intervention duration was 3 years, starting 6-12 months after adjuvant chemotherapy. Given the notable survival advantage and the general benefits of exercise in older adults, survivorship care should include early and feasible access to exercise programs.

Other important areas to improve survivorship care for older adults should be considered: Focus on nutrition and prevention of sarcopenia,^16^ incorporation of geriatric assessment into care planning, focus on care coordination, engagement of survivors and their caregivers in care provision, improving access to primary care,^17^ deprescribing of polypharmacy with medical rationalization, and improved provision of social support and social participation for older adults have been shown to improve mental health and address loneliness.^18^^,^^19^

This was a well-conducted study reporting on the real-world outcomes of a large population sample, but 3 limitations warrant specific focus. First, although internally valid, the requirements for robust cohort selection can create selection biases that ought to frame our reading of the results. As described earlier, the study population underrepresented vulnerable populations, whose unmet needs are not captured in this analysis. Further research should focus on the survivorship outcomes and care needs of vulnerable, older cancer survivors.^17^ Second, retrospective analyses are inherently unable to provide contemporaneous information about the needs of older breast cancer and CRC survivors today. Substantial changes to the treatment of breast cancer and CRC since the study period likely alter the long-term recurrence risk of future patients. Given incremental improvements in cancer treatments, however, the finding that recurrence risk is low in patients treated in the early 2000s ought only to underscore the implications of the findings for the long-term survivorship care of older adults today. Finally, real-world evidence cannot fully capture patterns of recurrence and other salient contributors to long-term health status and recurrence risk, such as body mass index, smoking status, and exercise habits.

Survivorship care must offer a clear value proposition for older adult long-term survivors of breast cancer and CRC. The study by Yasin et al.^3^ demonstrates that in a relatively continuously and well-insured cohort of older adults, cancer recurrence is not the dominant driver of mortality. In the absence of robust, evidence-based survivorship guidelines for this population, these findings suggest that care should prioritize interventions that improve global health and function, including promoting physical activity and nutrition, support for mental health, and strategies to mitigate frailty. Future research should focus on the needs of underrepresented and more vulnerable populations and translate these insights into scalable, equitable models of survivorship care for older adults.

## Data Availability

No new data were generated or analyzed for this editorial.
